# Remodeling Matrix Synthesis in a Rat Model of Aortocaval Fistula and the Cyclic Stretch: Impaction in Pulmonary Arterial Hypertension-Congenital Heart Disease

**DOI:** 10.3390/ijms21134676

**Published:** 2020-06-30

**Authors:** Chi-Jen Chang, Chung-Chi Huang, Po-Ru Chen, Ying-Ju Lai

**Affiliations:** 1 Cardiovascular Division, Chang Gung Memorial Hospital Chang Gung University, Linkou, Tao-Yuan 33353, Taiwan; chijenformosa@gmail.com; 2 Department of Respiratory Therapy, Chang Gung University College of Medicine, Tao-Yuan 33353, Taiwan; cch4848@adm.cgmh.org.tw; 3 Division of Thoracic Medicine, Chang Gung Memorial Hospital, Linkou, Tao-Yuan 33353, Taiwan; 4 Graduate Institute of Clinical Medical Sciences, Chang Gung University College of Medicine Tao-Yuan 33353, Taiwan; lulu122095@gmail.com; 5 Department of Respiratory Care, Chang-Gung University of Science and Technology, Chia-Yi 61363, Taiwan

**Keywords:** smooth muscle cell, cell stretch-induced response, volume-overloading induced pulmonary hypertension

## Abstract

Pulmonary arterial hypertension-congenital heart disease (PAH-CHD) is characterized by systemic to pulmonary arterial shunts and sensitively responds to volume overload and stretch of the vascular wall leading to pulmonary vascular remodeling. We hypothesized that the responses of pulmonary artery smooth muscle cells (PASMCs) to mechanical stress-associated volume overload may promote vascular remodeling in PAH-CHD. Here, we show that significantly increased collagen was in the PA adventitial layer by trichrome staining in PAH-CHD patients and an aortocaval fistula (ACF) rat model in which chronic vascular volume overload induced-PAH. We assessed the gene expression profiles of SMC markers, extracellular matrix, and collagen in isolated SMCs from pulmonary and thoracic vessels with cyclic stretch-triggered responses by real-time PCR analysis. The data corresponded to collagen deposition, which modulated pulmonary vascular remodeling in clinical and experimental PAH-ACF cases as well as in cyclic stretch-triggered SMCs in an in vitro model. We observe that collagen I A2 (COLIA2) is expressed in the control rat, but collagen I A1 (COLIA1) and Notchs remarkably increase in the lungs of ACF rats. Interestingly, closing the left-to-right shunt that leads to a reduced blood volume in the PA system of ACF rats (ACFRs) decreased the expression of COLIA1 and increased that of collagen I A2(COLIA2). This study contributes to the stretch-induced responses of SMCs and provides important future directions for therapies aimed at preventing abnormal matrix protein synthesis in volume overload-induced pulmonary hypertension (PH).

## 1. Introduction

The pulmonary circulation is a relatively low pressure system (approximately 14 mm Hg at rest) compared to the systemic circulation (approximately 100 mmHg at rest) because the pulmonary arteries (PAs) are not as muscularized as their systemic counterparts. In the systemic circulation, the artery is able to tolerate over 100 mmHg pressure, but in the pulmonary circulation, an arterial pressure increase to 20 mmHg can cause pulmonary vascular remodeling. Patients with congenital heart disease (CHD) have a central shunt between the systemic and pulmonary circulation (atrial and ventricular septal defects or patent ductus arteriosus), leading to volume overload and stretching of the pulmonary circulation. More than 90% of these patients are subjected to percutaneous or surgical repair early in life for a simple, noninvasive diagnostic evaluation [1,2]. In these instances, the clinical history, physical examination, chest X-ray, and echocardiographic analysis show features of a typical hyperkinetic pulmonary circulation associated with congestive heart failure, pulmonary congestion, and failure to thrive [3,4]. However, a probable 5%–10% of patients do not present with these features, even in the presence of unrestricted communications [2,3,4,5]. These patients are commonly subjected to invasive diagnostic examination (cardiac catheterization) prior to surgery, and a pulmonary vascular resistance (PVR) above 3 Wood units is detected as pulmonary arterial hypertension-congenital heart disease (PAH-CHD) [2,3]. Although all unrestricted communications may be associated with PAH, early development of pulmonary vasculopathy is particularly common in patients with irregularities, such as truncus arteriosus, transposition of the great arteries with a ventricular septal defect (VSD), and complete atrioventricular septal defects. Nearly 100% of unoperated patients with volume overload develop pulmonary hypertension (PH) [2,3,6].

Furthermore, PAH is characterized by increased PVR and caused by remodeling of the PAs [7,8]. Clinically defined PH requires an increase in the mean PA pressure of more than 20 mmHg [3,9,10]. The arteries in the lung create increased resistance to blood flow and blood pressure, increasing the right ventricular pressure, and thus, the workload of the heart. The important issue of PA pressure from rising above normal levels can be attributed to vasoconstriction, remodeling of the PA vessel wall, abnormal assembly of extracellular matrix (ECM) and thrombosis, leading to increased PVR in PAH [7,11,12]. Endothelial cells, smooth muscle cells (SMCs) and fibroblasts, as well as inflammatory cells and platelets, may play significant roles in PAH [7,8,13]. The cellular microenvironment provides critical input as cells work to achieve and maintain cellular homeostasis. In particular, ECM proteins in the microenvironment regulate a variety of processes, such as proliferation, migration, apoptosis, and the response to cellular signaling stimulation [14]. Vascular remodeling in PAH is characterized by the growth of neointimal lesions containing SMCs expressing ECM genes to contribute to abnormal elastic assembly and collagen deposition [15,16,17,18]. Elastic fibers are comprised of an insoluble elastin core and the surrounding mantle of the microfibrillar scaffold, deposition and integration of tropoelastin monomers into the scaffold, and cross-linking of the monomers to form an insoluble, functional polymer [16]. Arteries, for example, have more collagen and elastin than veins. Outside the smooth muscle cell (SMC) layer of large vessels is an adventitial layer extending beyond the external elastic laminae and interstitial matrix that contains fibrillar type I and III collagen, chondroitin sulfate and dermatan sulfate proteoglycans, fibronectin, and many other ECM proteins [19]. Moreover, increased collagen IA1 (COL1A1) leads to medial fibrosis and systemic vascular stiffening [20]. In gamma-irradiated fibroblasts, Komarova et al. showed that COL1A2 was increased in a p53-dependent manner and functions as a growth repressor, characterizing the "bystander effect" of p53-dependent gene therapy [21]. Chronic hypoxic exposure induces changes in the structure of PAs and triggers the TGF-beta signaling pathway, leading to increased elastolytic activity and collagen deposition [11,13,22,23].

Elastin and collagen are proteins for elasticity and play an important role in the regulation of vascular tone. Elastic fibers are an abundant and integral part of many extracellular matrices, in which they provide resilience and deformability to tissues [11,19,24]. These properties are critical for the functioning of tissues such as arterial vessels, lungs, and skin. Elastic fibers composed of two distinct components: elastin, an insoluble polymer of 70-kDa tropoelastin monomers, and microfibrils, 10-nm unbranching fibrillin-containing fibrils [19,24]. During development, the microfibrils form linear bundles that appear to act as scaffolds for the deposition and/or orientation of tropoelastin monomers. Lysine residues on adjacent monomers are then modified by the enzyme lysyl oxidase to form covalent cross-linking molecules, two of which (desmosine and isodesmosine) are unique to elastin and can be distinctive markers of the fully cross-linking insoluble fiber [19,25]. Electron microscopic analysis of lung biopsy specimens from patients with connective tissue disorders showed fragmented elastin in the PAs as an early feature of PAH [26]. Fragmentation of elastin can be the result of impaired assembly, increased enzymatic degradation or both [27]. In rodents with PAH induced by monocrotaline (MCT), the impaired assembly of newly synthesized elastin is linked to the heightened activity of elastases in the PA wall [28,29]. Increased activity of this collagen precedes the development of PAH and accompanies its progression. When the activity of these elastases was inhibited in a variety of rodent models, studies report attenuating both the hemodynamic and structural features associated with PAH [17,18,30]. Furthermore, studies have shown that the elastase activity can also induce regression of PAH by causing apoptosis of SMCs [17,18].

However, the role of the dynamic stretch link with ECM regulation is still not clear in the vascular biology of PA remodeling and vascular tone. Although advances in our understanding of the pathophysiology and the management of pulmonary hypertension [12], significant therapeutic gaps remain for this irreversible disease. Moreover, there are innate restrictions of the currently available in vitro and animal models, which unsuitably mimic the full spectrum of human pulmonary hypertension diseases [31]. This work was derived from studies in aortocaval fistula (ACF) rat models to understand the role of volume overload in PAH (ACF-induced PAH). Furthermore, the purpose of this study was to investigate the cellular and molecular changes in the regulation of ECM (e.g., collagen and elastin) in response to dynamic stretch and to review our current understanding of the mechanisms of collagen regulation in the phenotype of pulmonary vascular cell walls and vascular remodeling.

## 2. Results

### 2.1. Pulmonary Arteries of Patients with PAH-CHD Are Characterized by Collagen Upregulation and Elastic Laminae Degradation

**Figure 1:**
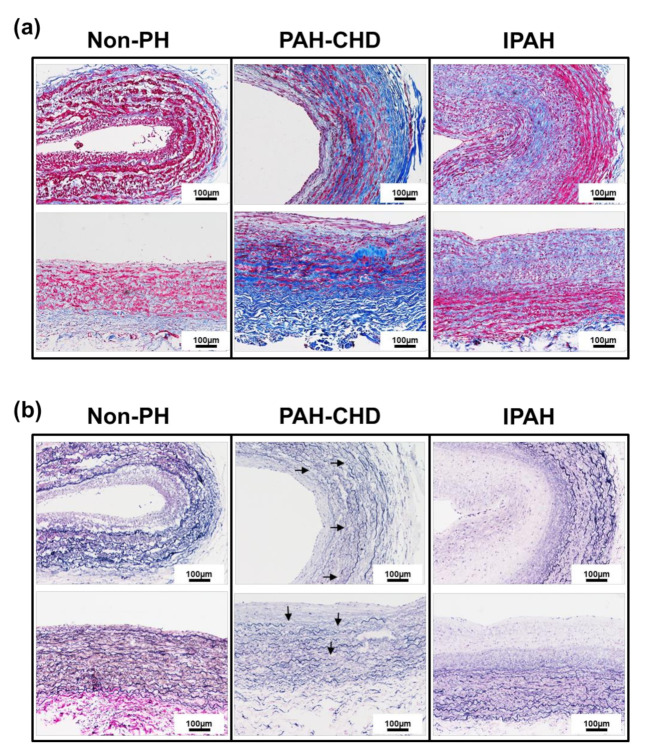
Collagen and elastin fiber distribution in the PAs of non-PH, PAH-CHD and IPAH patients. (**a**) Representative the Masson’s trichrome collagen staining in the pulmonary arteries PAs from the non-PH, PAH-CHD and IPAH patientpatients. The collagen content in the PA tissue from a PAH-CHD patient was significantly increased in the medial and adventitial layers compared with that in tissues from IPAH and non-PH patients. (**b**) Representative modified Verhoeff elastin-stained PAs in these three groups. Internal and external elastic laminae (black staining) are apparent in the non-PAH PA patient, but loss of elastin and fragmentation (arrows) are observed in the PAH-CHD patient (Scale bar: 100 µm).

In PA tissues of non-PH, PAH-CHD, and idiopathic pulmonary arterial hypertension (IPAH) patients, we investigated PA remodeling and the regulation of ECM (e.g., collagen and elastin). A histology experiment detected abundant collagen distribution in the medial layer and in the adventitia layer of PAH-CHD patients but not in IPAH patients. In contrast, collagen was less detectable in the PAs of the non-PH patients than in those with PH diseases (Figure 1a). We also observed structural changes in the elastic laminae of PAs. Modified Verhoeff staining showed extensive fragmentation in the elastic laminae in the PAs of non-PH, PAH-CHD and IPAH patients (Figure 1b). Significantly more elastic laminae fragments (Figure 1b) were observed in the PA tissues of patients with PAH-CHD than in the tissues of non-PH and IPAH groups. The abundant collagen distribution and impaired synthesis of tropoelastin or heightened production of poorly assembled elastin increases PA stiffness and resulted in fibers that are susceptible to cellular and molecular changes in the regulation of ECM (e.g., collagen and elastin) in response to inducing volume overload and stretching of the pulmonary circulation. The findings showed that the PAs of patients with PAH-CHD were characterized by collagen accumulation and elastic laminae degradation in the medial and adventitial layers.

### 2.2. Gene Profile of SMC Markers, Extracellular Matrix and Collagen in SMCs from Pulmonary and Thorax Vessels

**Figure 2:**
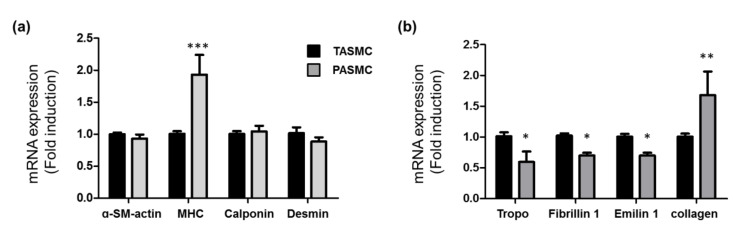
The SMC markers and ECM gene expression profiles of SMC. Representative qRT-PCR analysis. (**a**) The mRNA expression levels of SMC marker gene profiles. (**b**) ECM gene expression profiles. The expression differences were compared with GAPDH as a loading control. The SMCs were harvested for RNA at passage 1. Data are shown as the mean ± SEM in the same group of four individual SMCs isolated from either TASMCs or PASMCs. The black bars represent the TASMCs. The gray bars represent the PASMCs. * *p* < 0.05, ** *p* < 0.01, *** *p* < 0.001 compared with the TASMCs.

In the systemic circulation, artery is able to tolerate over 100 mmHg pressure, but in pulmonary circulation, artery increase to 20 mmHg can caused the pulmonary vascular remodeling [3,4]. Experimental evidence has demonstrated that the arterial media in both pulmonary and systemic vessels is heterogeneous in SMCs [32]. Our purpose was to isolate and maintain arterial SMCs in culture to observe the gene that correlated with changes in vascular pressure stretch. SMCs were isolated from the pulmonary and thoracic vessels of Sprague–Dawley (SD) rats. The SMC marker gene expression profiles were compared in the two groups of SMCs (Figure 2a). The mRNA expression was separately analyzed in four individual rats of each group, and this revealed variability in the patterns of gene expression and the patterns associated with the pulmonary circulation and the systemic circulation. The gene expression profiles of ECM and collagen in these two groups were also determined (Figure 2b). Within these two SMC groups (pulmonary circulation and systemic circulation), the expression of a-SM-actin, calponin and desmin was not different. Myosin heavy chain (MHC) in the PASMC group had significantly higher expression than MHC in the thoracic aorta SMC (TASMC) group. SM myosin can serve as an important marker of the SMC differentiation state [32]. In the ECM gene, real-time PCR analysis showed a decrease in the mRNA expression of ECM (tropoelasin, firillin 1, and emilin 1), and the expression of collagen I mRNA was higher in PASMCs than in TASMCs, suggesting that SMCs in PAs are differentiated and have less elasticity than cultured SMCs (Figure 2b).

### 2.3. Cyclic Mechanical Stretch Affects Collagen Expression Specifically in PASMCs

**Figure 3:**
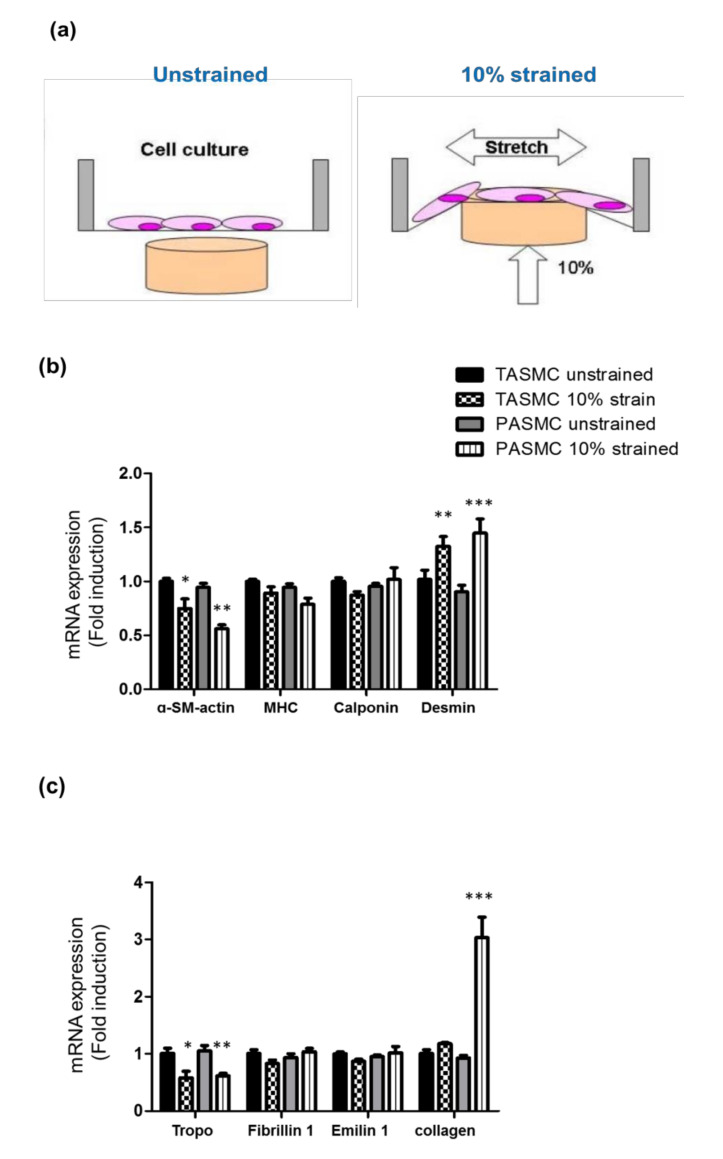
The gene profile of the TASMC and PASMC response to cyclic mechanical stretch. (**a**) A schematic description of the InVitro cyclic stretch model to mimic the experimental model of volume overload. Representative qRT-PCR analysis. The mRNA expression levels of (**b**) SMC markers or (**c**) the ECM gene profile from either TASMCs or PASMCs under 0% or 10% cyclic mechanical stretch. The expression differences were compared with GAPDH as a loading control. Data are shown as the mean ± SEM in the same group of four individual cell lines. The bars represent the means ± SEMs of four samples in each group. * *p* < 0.05, **p < 0.01 and *** *p* < 0.001 compared with the unstrained group.

Based on the gene profile of SMC markers, ECM and collagen, we observed these markers in SMCs. To better understand the mechanisms underlying the regulation of ECM assembly in the vascular stiffness of PAH in the dynamic increased blood flow of systemic and pulmonary circulation, we isolated and maintained arterial SMCs (TASMCs and PASMCs) to observe gene regulation response to the vascular pressure change and stretched the vascular wall by using the cyclic mechanical stretch model (Figure 3a). We aimed to test the hypothesis that vascular dynamic stretch progresses through the regulation of ECM deposition in PASMCs. SMC markers (a-SM-actin, MHC, calponin and desmin) and the relative ECM gene expression (troproelasin, fibrillin, emilin and collagen) were compared in four conditional groups of SMCs (Figure 3b,c): unstrained and 10% strained TASMCs and unstrained and 10% strained PASMCs. The mRNA expression was separately analyzed in four individual experiments in each group of SMCs, and this revealed variability in the pattern of gene expression and the pattern associated with dynamic increased blood flow of systemic and pulmonary circulation. Quantification of the SMC marker and the ECM relative gene expression of these four groups was performed and set the unstrained group as 1-fold induction and compare the gene expression between unstrained group and strained group (Figure 3b,c). The data are shown as the mean ± SEM for the same group of four individual cell lines isolated from rat PASMCs and TASMCs. Within these four cell conditional groups, MHC and calponin were unchanged, a-SM-actin and tropoelasin were downregulated, and desmin was upregulated in both the 10% strained TASMC and PASMC groups compared with those in the unstrained groups. Interestingly, out of all the groups, the expression of collagen was upregulated in only the 10% strained PASMC group. To the best of our knowledge, these findings are the first to identify that the gene expression to respond in the cyclic mechanical stretch model exhibits different behavior in TASMCs and PASMCs.

### 2.4. ACF Impacts Right Heart Hypertrophy and the Overexpression of Collagen, Leading to PA Stiffness

**Figure 4:**
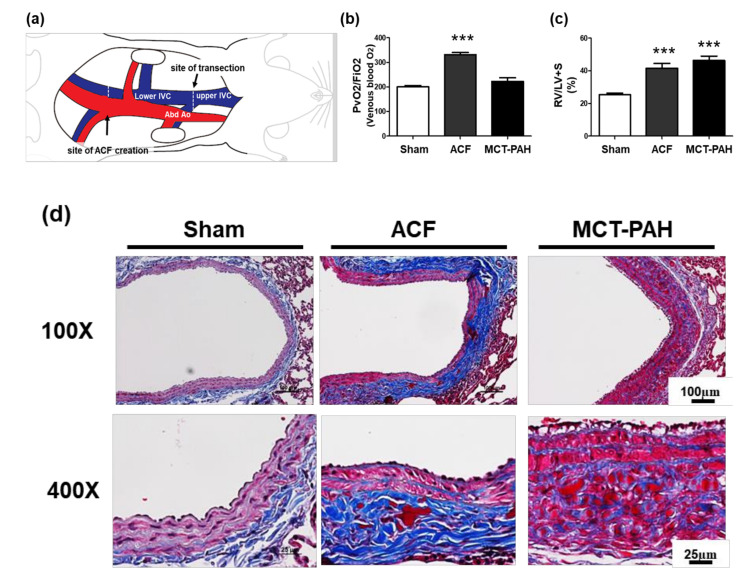
Characterization of the ACF animal model: ACF impacts right heart hypertrophy and collagen accumulation, leading to PA fibrosis. (**a**) A schematic description of the creation of ACF in rats, an experimental model of volume-overload CHF. (**b**) Oxygenation index of the IVC in the sham, ACF and MCT-PAH groups. (**c**) The ratio of RV to LV plus septum weight (RV/LV+S) is shown. Means ± SEMs, *** *p* < 0.001 versus sham. (**d**) Representative Masson’s trichrome collagen staining in the PAs from the sham, ACF and MCT-PAH rat models. The PA tissue from an ACF model had significantly increased collagen accumulation in the adventitial layers compared with that in the sham and MCT-PAH models.

To pursue our observation that identifies whether collagen deposition is abnormal in the PAs of patients with PAH-CHD and in vitro cyclic mechanical stretch models, we established a flow-induced PH animal model with ACF surgery in SD rats [33], observed the morphological changes and studied the altered histological and molecular events in PA (Figure 4a). The lower lumbar aorta was punctured with a needle. The needle was inserted into the lateral wall of the aorta to cross the vascular wall of the inferior vena cava (IVC). To evaluate successful aortocaval fistula surgery, we observed that venous oxygenation in ACF rat (ACF) models was significantly increased compared with that in sham or MCT-induced PAH models (Figure 4b). In the well-known MCT-induced PAH groups, significant RV hypertrophy developed as a consequence of increased pulmonary pressures. The ratio of RV weight to LV plus septum weight (RV/LV+S) increased from 25.31% (sham) to 41.52% (flow-induced pulmonary hypertension ACF animal model) and 46.3% (28 days after MCT challenge) (both ***p < 0.05 versus controls) (Figure 4c). Collagen deposition occurred in the external media and adventitia of the ACF PA. Masson’s trichrome staining showed increased collagen in the ACF PA external media layer. The medial abnormality morphology is different in the pulmonary trunk of ACF and MCT-induced PAH animals (Figure 4d). Consistent with the clinical and in vitro findings, we found that PA tissue from an ACF rat model had significantly increased collagen accumulation in the adventitial layers compared with the PA tissue from the sham and MCT-PAH models, highlighting the critical role of collagen in the flow-overloading development of PA fibrosis.

### 2.5. Effect of Flow Stress on Notch and Collagen Activation in the Pulmonary Circulation

**Figure 5:**
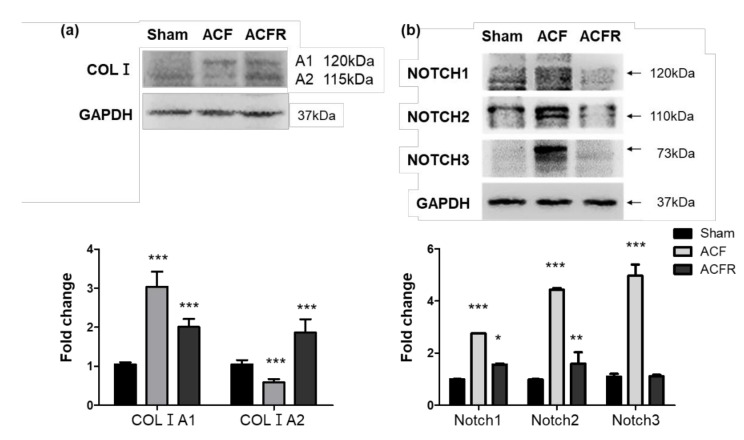
Effect of dynamic stretch on collagen IA2 to A1 isoform and Notch3 activation in ACF animal model. After 4 months of ACF, we then closed the aortocaval fistula for one month (ACFR). (**a**) Western blot showing collagen IA2 to A1 expression in total lung cell lysis. The relative expression level of each protein was quantified by densitometry and normalized to the expression level of GAPDH. (**b**) Western blot showing Notch1, 2, and 3 expression in total lung cell lysates. The relative expression level of each protein was quantified by densitometry and normalized to that of GAPDH. Each value represents the mean ± SE of 4 independent experiments. * *p* < 0.05, ** *p* < 0.01 and *** *p* < 0.001 versus sham group, one-way ANOVA, Dunnett’s post test.

Arteries have more collagen and elastin than veins [19]. An adventitial layer exists outside the SMC layer of large vessels, extends beyond the external elastic laminae and interstitial matrix and contains fibrillar type I and III collagen, chondroitin sulfate and dermatan sulfate proteoglycans, fibronectin, and many other ECM proteins [19]. Increased collagen I A1 (COLIA1) leads to medial fibrosis and aortic stiffening [20]. In gamma-irradiated fibroblasts, Komarova et al. showed that collagen I A2 (COLIA2) was increased in a p53-dependent manner, functions as a growth repressor, and characterizes the "bystander effect" of p53-dependent gene therapy [21]. Chronic hypoxic exposure induces changes in the structure of PAs and triggers the TGF-beta signaling pathway, leading to increased elastolytic activity and collagen deposition [13,22,23]. The Notch signaling pathway is activated by gamma-secretase cleavage and the ensuing release of its ICD fragments into the nucleus and promotes PAH [34,35]. We then observed the relative proteins that responded to flow-induced vascular overloading. COLIA1 and COLIA2 reflect a dynamic adjustment in response to the stress of volume overload but not tropoelasin. Western blot analysis using whole lung lysates showed that COLIA2 was expressed primarily in the sham group. With dynamic stretch overload, COLIA1 and Notch1–3 were increased in the ACF group. Interestingly, closing the left-to-right shunt that leads to a reduced blood volume in the PA system decreased Notch1, 2, and 3. Moreover, there was decreased COLIA1 but increased COLIA2 in the ACFR group (Figure 5a,b). COLIA2 and COLIA1 isoforms are formed in response to dynamic stretch overload in vivo. Taken together, these findings demonstrate that dynamic stretch stress may affect Notch activation and the collagen I isoform in the lungs of ACF.

## 3. Discussion

Patients with CHD have simple communications between the systemic and pulmonary circulation, atrial and ventricular septal defects, or patent ductus arteriosus that induce flow overloading and vascular stretch stress, leading to PA remodeling and stiffness. PAH-CHD is an early phase in which arteriopathy can generally be reversed by closing communication between the systemic and pulmonary circulation to reduce volume-overload stress. In this study, we propose to observe and study the pathophysiological and molecular mechanisms in clinical PAH-CHD and establish an ACF animal model, as well as a dynamic stretch in vitro model. These studies elucidate the relative importance of downstream signaling pathways underlying the dynamic stretch effects of collagen signaling transduction. Moreover, it is of interest to profile the downstream target factors due to the dynamic volume-overload stretch effect in the ECM assembly of vascular SMCs under investigation to clarify the pathology of PAH-CHD. In this study, a PAH-CHD patient had significantly increased collagen accumulation in the adventitial layer than an IPAH patient. We further confirmed collagen deposition in the PA wall through an ACF model in which chronic vascular volume overload induced PAH. The data relate to collagen deposition in clinical and experimental ACF-PAH as well as in a cyclic stretch-triggered SMC in vitro model. We show the overexpression of collagen I (COLIA1) and Notchs in the lungs of ACF rats. Interestingly, closing the left-to-right shunt that leads to a reduced blood volume in the PA system of ACF (ACFR) decreased the expression of COLIA1 and increased COLIA2. This study contributes to the stretch-induced responses of SMCs and provides important future directions for therapies aimed at preventing abnormal matrix protein synthesis in volume overload-induced PH.

Recent studies state survival in PAH as disease stability rather than disease reversal. In idiopathic PAH, the trigger cannot be removed, and vascular morphology is normalized. However, this unique type distinguishes PAH-CHD from other groups of PH, and arteriopathy can generally be reversed by closing the communications between the systemic and pulmonary circulation to reduce volume-overload stress and result in full disease regression [36,37]. In PAH-CHD, remodeling of the pulmonary vasculature reaches an irreversible phenotype similar to all forms of end-stage PAH. In our studies, for the first time, to compare the PA histology of IPAH and PAH-CHD, we investigated PA remodeling and the regulation of ECM (e.g., collagen and elastin). The histology study detected more abundant collagen distribution mainly in the adventitia layer of PAH-CHD compared to the collagen distribution in IPAH. We also observed structural changes in the elastic laminae of PAs. Modified Verhoeff staining showed extensive fragmentation in the elastic laminae in the PAs of PAH-CHD and IPAH patients. A significant number of elastic laminae fragments were found in the PA tissues of patients with PAH-CHD. The abundant collagen distribution and impaired synthesis of tropoelastin or heightened production of poorly assembled elastin increases PA stiffness and results in fibers that are susceptible to the cellular and molecular changes in the regulation of the ECM (e.g., collagen and elastin) in response to induced volume overload and pulmonary circulation expansion. We aimed to pursue our observation that identifies whether collagen deposition is abnormal in the PAs of patients with PAH-CHD, and we established an ACF and MCT rat model to observe the pathohistological changes and study the altered molecular events in the histology of PA. To evaluate the successful aortocaval fistula model, we demonstrated that venous oxygenation in ACFR models was significantly increased compared with that in sham or MCT-induced PAH. In the MCT-induced PAH groups, significant RV hypertrophy developed as a consequence of increased pulmonary pressure. RV hypotrophy is caused in both the ACF animal model and MCT-induced PAH. However, collagen deposition in the adventitia of the ACF PA is completely different from that in the MCT-induced PAH model. This is consistent with the clinical and in vivo findings that the PA tissue from the PAH-CHD and ACF models revealed significantly increased collagen accumulation in the adventitial layers compared with the tissue from the IPAH and MCT-PAH models, highlighting the critical role of collagen in the flow-overloading development of PA fibrosis.

However, the arteriopathy of PAH-CHD can generally be reversed by closing the communication shunt between the systemic and pulmonary circulation to reduce the volume-overload stress and result in full disease regression. COLIA1 and COLIA2, the relative proteins that respond to flow-induced vascular overloading, reflect a dynamic adjustment in response to the stress of volume overload but not of tropoelasin. COLIA2 was expressed primarily in the normal vascular environment. Upon dynamic stretch overload, COLIA1 and Notch1-3 were increased in ACF rat. Closing the left-to-right shunt that leads to a reduced blood volume in the PA system (ACFR rats) decreased Notchs and decreased COLIA1 but increased COLIA2. For the first time, we report a dynamic switch in the COLIA2 and COLIA1 isoforms in response to flow-overload stress stretching in vivo. There is an adventitial layer outside the SMC layer of large vessels, and this layer extends beyond the external elastic laminae and interstitial matrix that contains fibrillar type I and III collagen, chondroitin sulfate and dermatan sulfate proteoglycans, fibronectin, and many other ECM proteins [19]. COLIA1 has been reported to affect medial fibrosis and aortic stiffening [20]. In gamma-irradiated fibroblasts, Komarova et al. showed that COLIA2 was increased in a p53-dependent manner, functions as a growth repressor and characterizes the "bystander effect" of p53-dependent gene therapy [21]. Chronic hypoxic exposure induces changes in the structure of PAs and triggers the TGF-beta signaling pathway, leading to increased elastolytic activity and collagen deposition [13,22,23]. The Notch signaling pathway is activated by gamma-secretase cleavage and the ensuing release of its ICD fragments into the nucleus and promotes PAH [34,35]. In this study, the mRNA level of Notchs did not change under the in vitro stretching experiments, but in the in vivo ACF and ACFR models, we detected changes in the Notch 1–3 protein levels. Notch signaling is regulated at multiple levels, including the patterns of ligand and receptor expression, Notch–ligand interactions, trafficking of the receptor and ligands, and covalent modifications, including glycosylation, phosphorylation, and ubiquitination [38,39]. A possible mechanistic explanation for the effect of cyclic stretch on Notch activity may be posttranslational modifications that may influence its transactivation capacity. However, the limitation of this study is that the area of the stretch membrane is too small to accumulate proteins from cells. We were not able to observe protein level changes. Mechanistically too, the relevance of the elevated levels of Notch 1, 2, and 3 are not able to made clear. Taken together, these findings demonstrate that dynamic stretch stress may affect Notch activation and collagen I isoforms in the lungs of ACF rats. In clinical studies, arteriopathy can generally be reversed by closing the communication shunt to reduce volume-overload stress and result in full disease regression [36,37]. Unfortunately, the proportion of PAH-CHD with disease progression is still significant despite early surgery [40]. Moreover, 5%–13% for adults [41,42]. In PAH-CHD patients, older age at correction is an important factor for irreversible disease [9]. This study has shown that vascular stiffness by COLA1 accumulation and elastic laminae degradation are still in the PAs and have a long-term impact on the development of PAH-CHD.

In the systemic circulation, the artery is able to tolerate over 100 mmHg pressure, but in the pulmonary circulation, a pressure increase in the artery to 20 mmHg can cause pulmonary vascular remodeling [3,4]. Experimental evidence has demonstrated that the arterial composition of both the pulmonary and systemic vessels consists of heterogeneous SMCs and ECM regulation in the adventitia layer [32]. In this study, for the first time, we established two isolated populations of arterial SMCs to observe the gene that correlated with the vascular pressure change. The SMC marker gene expression profiles were compared in the pulmonary and thorax vessel groups of SMCs to reveal variability in the pattern of gene expression associated with pulmonary circulation and systemic circulation. Within the two SMC groups (TASMCs and PASMCs), the expression of a-SM-actin, calponin and desmin was not different. MHC expression was significantly higher in the PASMC group than in the TASMC group. SM myosin can serve as an important marker of the SMC differentiation state [32]. In the ECM gene, real-time PCR analysis showed a decrease in the mRNA expression of ECM (tropoelasin, firillin 1, and emilin 1), and the mRNA expression of collagen I was higher in PASMCs than in TASMCs, suggesting that PASMCs are more differentiated and less elastic than cultured TASMCs.

The normal contractile type of SMCs can adjust myogenic tone, to control blood pressure, and response to blood flow within the blood vessel [43]. Conversely, in the environment to respond to vascular injury, vascular smooth muscle cells (VSMCs) often shift from a contractile to a synthetic, or undifferentiated, phenotype, which is characterized by a decrease in the expression of contractile markers. Moreover, synthetic VSMCs display increased rates of VSMC proliferation, migration, and ECM remodeling [32,44]. In 2015, Mantella et al. reviewed VSMCs under in vitro conditions to react to mechanical stress [45]. In those studies, major variances were noted in mechanical stress-induced proliferation, apoptosis, and phenotypic switching responses, depending on the stretch conditions. However, these discrepancies derive from variations in stretch conditions such as degree, axis, duration, and frequency of stretch; wave function; membrane coating; cell type; cell passage number; culture media content; and choice of in vitro model [45]. In this study, for the first time, we established two isolated populations of arterial SMCs to observe the gene that correlated with the vascular pressure change. Within the two SMC groups (TASMCs and PASMCs), the expression of a-SM-actin, calponin and desmin was not different. MHC expression was significantly higher in the PASMC group than in the TASMC group. Based on the gene profile of SMC markers, ECM and collagen, we observed these markers in SMCs. To better understand the mechanisms underlying the regulation of ECM assembly in vascular stiffness of PAH in dynamic increased blood flow of systemic and pulmonary circulation, we then used the cyclic mechanical stretch model to test the hypothesis that vascular dynamic stretch progresses through the regulation of ECM deposition in PASMCs. SMC markers and the ECM relative gene expression with unstrained and 10% strained TASMCs or unstrained and 10% strained PASMCs were used. Within these four cell group conditions, MHC and calponin were unchanged. Moreover, a-SM-actin and tropoelasin were downregulated and desmin was upregulated in both the 10% strained TASMC and PASMC groups compared with the unstrained groups. Interestingly, the expression of collagen was upregulated in only the 10% strained PASMC group compared with the unstrained groups or the 10% strained TASMC group. To the best of our knowledge, these findings are the first to identify that the gene present in the cyclic mechanical stretch model exhibits different behaviors in TASMCs and PASMCs. However, the limitation of this study is that the area of the stretch membrane is too small to accumulate proteins from cells. We were not able to observe protein level changes. Mechanistically too, the relevance of the elevated levels of Notch 1, 2, and 3 are not able to made clear.

The disturbance of collagen features is a typical part of PAH-CHF pathophysiology. This work was derived from studies in ACF rat models to understand the role of volume overload in PAH (ACF-induced PAH) and to understand the mechanisms of collagen regulation in the phenotype of pulmonary vascular cell walls and vascular remodeling. According to the literature data and our own results, this study contributes to the stretch-induced responses of SMCs and shows that vascular stiffness by COLA1 accumulation and elastic laminae degradation are still in the PAs and have a long-term impact on the development of PAH-CHD. This study provides important future directions for therapies aimed at preventing abnormal matrix protein synthesis in volume overload-induced PH.

## 4. Materials and Methods

### 4.1. Patient Characteristics

Human lung tissues were received from non-PH patients and patients with IPAH or PAH-CHD undergoing lung surgery at National Taiwan University Hospital. Informed consent was obtained from all individuals. The protocol for tissue collection was permitted by the Human Research Ethics Committee at National Taiwan University Hospital (Institutional Review Board 201409069RINA) and Chang Gung Memorial Hospital (Chang Gung Medical Foundation Institutional Review Board 104-0287B), and the study was conducted in accordance with the principles of the Declaration of Helsinki.

### 4.2. Rat Model of ACF

An ACF model [33] was developed in SD rats (300–350 mg) anesthetized with isoflurane (). The rats were placed in a supine position with 4 limbs fixed. After anesthetization with ketamine (50 mg/kg) and xylazine (8 mg/kg), the abdominal cavity was opened. The IVC and aorta were exposed, and a clamp was placed across both. The lower lumbar aorta was punctured with an 18-gauge needle. The needle was inserted into the lateral wall of the aorta, advanced medially to across the medial wall and the nearby wall of IVC, and then gently withdrawn. The entry point in the aortic wall was sealed with cyanoacrylate. The abdominal wall was closed, and the rats were allowed to recover. In the sham operation group, the same procedure was performed except that the needle did not puncture the medial wall of the aorta. After the operation, the rats were kept warm by light. One milliliter of 1% lidocaine was administered Intraperitoneal injection (i.p) pro re nata (PRN) to relieve pain, and 20 mg cefazoline was administered ip for prophylaxis. When the rats were sacrificed, ketamine (50 mg/kg) and xylazine (8 mg/kg) were administered i.p. for anesthesia. All animal experimental protocols were reviewed and approved by the Chang Gung University IACUC (Permit Number: CGU107-192, 2018 approved). This study was carried out in strict accordance with the recommendations in the Guide for the Care and Use of Laboratory Animals of the National Institutes of Health. Housing and maintenance were provided by Chang Gung University, and all animals were fed a standard chow diet with free access to water. The partial pressure of oxygen (PaO2) was measured by a blood gas analyzer to determine the oxygenation index (PaO2/FiO2).

### 4.3. Monocrotaline-Treated (MCT) Rats

Adult male SD rats (200–250 g in body weight) received either a subcutaneous injection of 60 mg per kg body weight of crotaline (Sigma, Saint Louis, MI, USA) or saline alone to induce PAH and RV hypertrophy after 4 weeks [46]. All institutional and national guidelines for the care and use of laboratory animals were followed, and the protocol was approved by the Chang Gung University institutional committees IACUC (permit number: CGU107-192, 2018 approved). Animal housing and maintenance were provided by Chang Gung University, and all animals were fed a standard chow diet with free access to water.

### 4.4. Isolation and Culture of Animal Arterial SMCs

The SMCs were isolated from SD rats, as described previously [47,48]. To obtain PA and thoracic arterial SMCs (PASMCs and TASMCs), the artery was dissected from the lung and cardiac tissues. The arterial tissues were then cut into small pieces. After approximately 72 h, SMCs started to migrate out from the tissues. Cells were resuspended in DMEM (GIBCO, Paisley, UK) supplemented with 100 U/mL penicillin and 100μ g/mL streptomycin (PAN), 0.5 mM l-glutamine (GIBCO, Paisley, UK), and 10% fetal calf serum and incubated at 37 °C in 5% CO_2_-95% air. The medium was changed then and thereafter every 2–3 days. To characterize SMCs, we used the smooth muscle cell–specific gene markers α-smooth muscle actin (Sigma-Aldrich, Saint Louis, MO, USA) and desmin (Abcam, Cambridge, MA,. USA). Desmin was down-regulated at passage 3. Therefore, PASMCs were used before passage 2 for all of the in vitro experiments. The SMCs were studied from passages one to two. SMCs were characterized by immunocytochemical staining for α-smooth muscle actin and desmin.

### 4.5. Mechanical Stretch Assay

Cyclical stretch-relaxation was applied for cultured PASMCs and TASMCs. Cells were grown to 80% confluence and cultured in DMEM with 0.5% fetal bovine serum on six-well silicone elastomer-bottomed culture plates (Bioflex; Flexcell, BF-3001C, Hillsborough, NC, USA) that had been coated with collagen type I. After incubating for 48 h in the quiescent medium, cells were subjected to cyclical stretch/relaxation using the Flexercell Strain Unit FX-4000 (Flexcell). This unit (Figure 3a) is a modified version of the one initially described by Wipff et al [49]. Under computer control, a vacuum (<15 to 20 kPa) was repetitively applied (60 cycle/min, the maximal stretch of 10% at the periphery) to the silicone elastomer-bottomed culture plates, which were maintained in a humidified incubator with 5% CO_2_-95% air atmosphere at 37 °C. Cells were subjected to stretch/relaxation for a variable duration up to 24 h as indicated.

### 4.6. Histological Examination and Morphometric Analysis

The technicians obtained 6 samples from each pulmonary artery. The sampling should target different areas within the arteries to obtain an adequate representation of the whole tissue. For all histological procedures, the tissue was fixed with 10% neutral buffered formalin, immersed in formalin overnight, and then embedded in paraffin. Sections (4 μm thick) were stained with hematoxylin and eosin or Masson’s trichrome stain or modified Verhoeff elastin stain for morphometric analysis. The staining was processed according to the manufacturer’s instructions (ScyTek, Logan, UT, USA).

### 4.7. Total RNA Extraction and Quantitative Real-Time Reverse Transcription-Polymerase Chain Reaction

To characterize the relative gene expression of SMCs, total RNA was isolated using the TRIzol method for real-time PCR analysis. The concentration and quality of RNA were determined by spectrophotometry (Nanodrop 2000C, Thermo, Wilmington, DE, USA). Complementary DNA was synthesized using 1 mg total RNA in a 20 μL volume RT reaction mix (Life Technologies, Carlsbad, CA, USA). Primers for quantitative real-time RT-PCR were designed with the Primer3 program (http://bioinfo.ut.ee/primer3/) (Table 1). Quantitative real-time PCR was completed using EvaGreen dye (Biotools, New Taipei City, Taiwan) and processed according to the manufacturer’s instructions. Aliquots (20 ng) of cDNA were added for each quantitative PCR, and each reaction was carried out in triplicate. Quantitative real time PCR was performed with universal cycling conditions (15 min at 95 °C, followed by 40 cycles of 30 s at 95 °C, 1 min at 56 °C, and 30 s at 72 °C). Cycle threshold (CT) values were determined by automated threshold analysis with Mx-Pro Mx3005P v4.00 software (Stratagene, La Jolla, CA, USA) and the expression levels of glyceraldehyde 3-phosphate dehydrogenase (GAPDH) were monitored as a loading control.

### 4.8. Immunoblotting

For the isolation of proteins from arterial tissues, the samples were lysed by the addition of SDS-PAGE sample buffer followed by homogenization. After transfer to PVDF membranes (Millipore, Darmstadt, Germany), proteins were incubated with primary antibodies using anti-collagen I (Abcam, ab21286, Cambridge, UK), NOTCH1, 2, and 3 (Cell Signaling, Danvers, MA, USA). Secondary antibodies specific for peroxidase-conjugated anti-mouse IgG (Sigma-Aldrich; 1:10,000, St. Louis, MO) or anti-rabbit IgG (Sigma-Aldrich; 1:5000, St. Louis, MO) were used as needed. Blots were visualized using an enhanced chemiluminescence detection system (Amersham, Buckinghamshire, UK). Samples were normalized to GAPDH (Santa Cruz; 1:10,000, SC-32233, CA, USA) and quantified by densitometry.

### 4.9. Statistical Analysis

Data are defined as the mean and standard error (SE). Differences between two groups were determined by an unpaired t-test. For multiple groups, one-way ANOVA with Bonferroni’s post hoc test was applied to compare the data. A value of *p* ≤ 0.05 was considered statistically significant.

## Figures and Tables

**Table 1:** Primer sequences used in real-time-polymerase chain reaction.

Primer Name	Sequence	Amplicon Size (bp)
α-SM-actin	5′-CTGCT GTCTC TGTCC TTCTA C-3′	151
	5′-CCGAAGGACA GACCA GAAAC-3′	
MHC	5′-CGACT TGACA ACCAA CCTA-3′	108
	5′-TTCTT CAGCC TCACC TCTA-3′	
Calponin	5′-CAAAGTTGCTTCCCAGAAAGG A-3′	161
	5′-TTCTG CCTGG TAATCGCTAT CA-3′	
Desmin	5′-GCAGG TCCAG GTAGA GAT-3′	97
	5′-GATGT TCTTA GCCGC AATG-3′	
GAPDH	5′ AGTCT ACTGG CGTCT TCA-3′	136
	5′- TTGTC ATATT TCTCG TGGT-3′	
Fibrillin1	5′-CGTAT CTCCA TTGTC TCCC-3′	112
	5′-ATGCG ACTGT CCACC TGA-3′	
tropoelastin	5′-TGTTG GAGTT GGCGG AGTT-3′	147
	5′-GGAAG ACCGA CACCA GGAA-3′	
Emilin-1	5′-AGGAC CGCTT CAACT CTACC-3′	185
	5′-TGCCA CCTGC TCTTC CAATA-3′	
Collage I	5′-TGACC GATGG ATTCC AGTTC-3′	132
	5′-GCTGT TCTTG CAGTG ATAGG-3′	

## Abbreviations

ACF	Aortocaval fistula
ACFRs	ACF rats
CHD	Congenital heart disease
COLIA1	Collagen I A1
COLIA2	Collagen I A2
ECM	Extracellular matrix
GAPDH	Glyceraldehyde 3-phosphate dehydrogenase
IACUC	Institutional animal care and use committee
IPAH	Idiopathic pulmonary arterial hypertension
i.p.	Intraperitoneal
IVC	Inferior vena cava
MCT	Monocrotaline
MHC	Myosin heavy chain
PAH-CHD	Pulmonary arterial hypertension-congenital heart disease
PaO2	Partial pressure of oxygen
PAs	pulmonary arteries
PASMCs	Pulmonary artery smooth muscle cells
PH	Pulmonary hypertension
PRN	pro re nata
PVR	Pulmonary vascular resistance
RV/LV+S	Right Ventricle weight to Left Ventricle plus septum weight
SE	Standard error
SD	Sprague-Dawley
SMC	Smooth muscle cell
TASMC	Thoracic aorta smooth muscle cell
VSD	Ventricular septal defect
VSMCs	Vascular smooth muscle cells
